# A Chromosomal Region on ECA13 Is Associated with Maxillary Prognathism in Horses

**DOI:** 10.1371/journal.pone.0086607

**Published:** 2014-01-21

**Authors:** Heidi Signer-Hasler, Markus Neuditschko, Christoph Koch, Sylvie Froidevaux, Christine Flury, Dominik Burger, Tosso Leeb, Stefan Rieder

**Affiliations:** 1 School of Agricultural, Forest and Food Sciences, Bern University of Applied Sciences, Zollikofen, Switzerland; 2 Swiss National Stud Farm, Agroscope, Avenches, Switzerland; 3 Institute of Genetics, Vetsuisse Faculty, University of Bern, Bern, Switzerland; 4 Swiss Institute of Equine Medicine ISME, Vetsuisse Faculty University of Bern and Agroscope, Bern, Switzerland; Instituto de Ciencia de Materiales de Madrid - Instituto de Biomedicina de Valencia, Spain

## Abstract

Hereditary variations in head morphology and head malformations are known in many species. The most common variation encountered in horses is maxillary prognathism. Prognathism and brachygnathism are syndromes of the upper and lower jaw, respectively. The resulting malocclusion can negatively affect teeth wear, and is considered a non-desirable trait in breeding programs. We performed a case-control analysis for maxillary prognathism in horses using 96 cases and 763 controls. All horses had been previously genotyped with a commercially available 50 k SNP array. We analyzed the data with a mixed-model considering the genomic relationships in order to account for population stratification. Two SNPs within a region on the distal end of chromosome ECA 13 reached the Bonferroni corrected genome-wide significance level. There is no known prognathism candidate gene located within this region. Therefore, our findings in the horse offer the possibility of identifying a novel gene involved in the complex genetics of prognathism that might also be relevant for humans and other livestock species.

## Introduction

Advances in genotyping, sequence analysis, and data-mining technology have enhanced genetic research in livestock species during the last decade. Genomic research in equids has also benefited from these developments [1], [2]. Several spectacular findings, such as the discovery of selection signatures [3], the description of diverse genes responsible for different coat color phenotypes [4], [5], diagonal and lateral locomotion patterns [6], racing performance [7]–[9], height and conformation traits [10]–[12], and hereditary disorders [13], [14], highlight the potential of these new technologies. Breed diversity studies, including population structure analyses represent another important research field that has significantly advanced during the last few years [15]. In addition to the identification of causative variants for a variety of phenotypes, the livestock industry is particularly interested in using genomic information for the estimation of breeding values [16]–[18].

**Figure 1:**
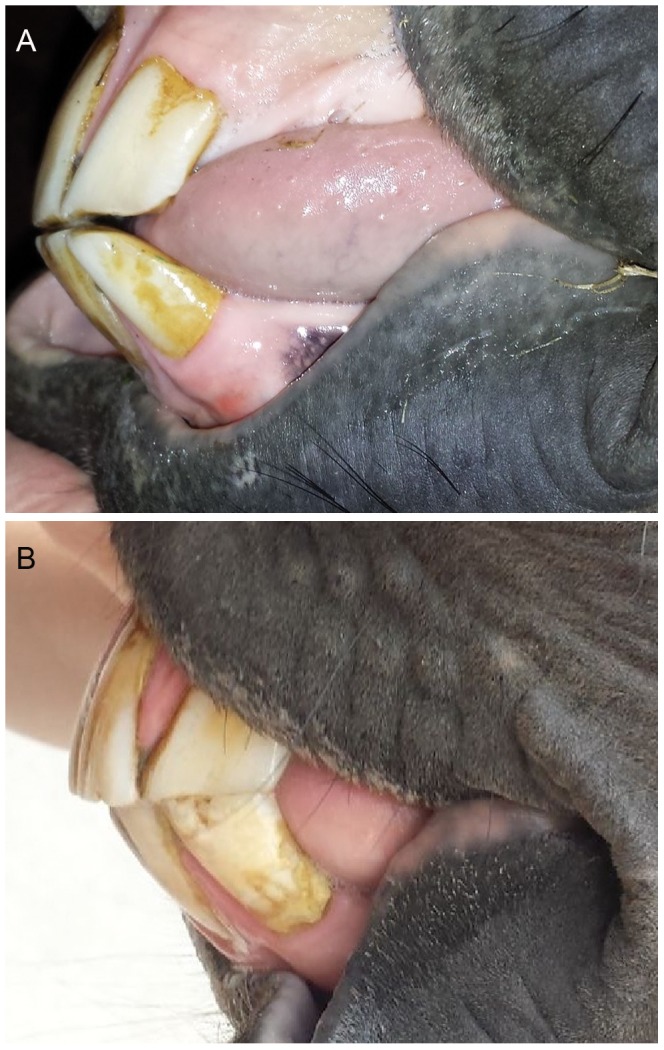
Maxillary prognathism phenotype. (A) Unaffected phenotype with normal occlusion. (B) Affected phenotype: moderate maxillary prognathism with resulting malloclusion of the incisor teeth (Picture: ISME).

Variations in skull morphology, such as an incompatible length of the upper jaw (maxilla) and lower jaw (mandible) may result in malocclusion of incisors as well as cheek teeth. Jaw malformations are widely known in mammalian species, including humans, and in vertebrates in general. It is usually hypothesized that environmental and genetic factors contribute to this syndrome. Although the exact etiology is unknown, the descriptive terminology referring to these variations often implies that one jaw is too long, prognathism, or the other too short, brachygnathism [19]. Consequently, terms describing the same phenomenon are often used interchangeably and inconsistently in the medical literature. In horses, the most commonly encountered condition is maxillary prognathism, also known as overjet or “Parrot Mouth” - Figure 1 [19]. The resulting malocclusion can negatively affect teeth wear and correct chewing movement, with the potential for diverse clinical consequences [19]. Furthermore, the syndromes may also have negative implications on the designated use (riding, driving) of an affected horse, as horses with malocclusion might be particularly uncomfortable with their bit. Thus, jaw malformations are considered non-desirable traits in domestic animal breeding programs, also relatively little is known about the hereditary background of the trait so far [14]. However, performing a clinical examination on 702 three year old Franches-Montagnes horses and 493 three year old Warmblood horses, the prevalence of maxillary prognathism was found 3.4% for FM and 8.5% for Warmblood horses, respectively [20], [21]. This is similar to the 2–5% prevalence of maxillary prognathism reported in other equine studies [22].

In a recent human study, matrilin 1 (*MATN1*) gene was found associated with mandibular prognathism [23].Using a candidate gene approach, Rodrigues and colleagues investigated malocclusions in an endangered Spanish donkey breed as a model species an reported an intronic variant in the *MATN1* gene. Statistically significant differences at this variant were found between the control group and the prognathism cases, but not between the control group and the brachygnathism cases [24].

Franches-Montagnes (FM) are a genetically closed and indigenous Swiss horse breed consisting of about 21,000 horses with 2,500 foalings per year [10], [25]. Here, we report the association of a chromosomal region on ECA 13 with maxillary prognathism in FM horses.

## Results

### Phenotype

The maxillary prognathism phenotype is shown in figure 1. Mold imprint measurements of horses without visual evidence of maxillary prognathism (n = 28) compared with those obtained from horses classified as being affected with maxillary prognathism (n = 32) showed that visual classification is valid (detailed results can be found in the supplementary figures 1–3).

### GWAS for maxillary prognathism

**Figure 2:**
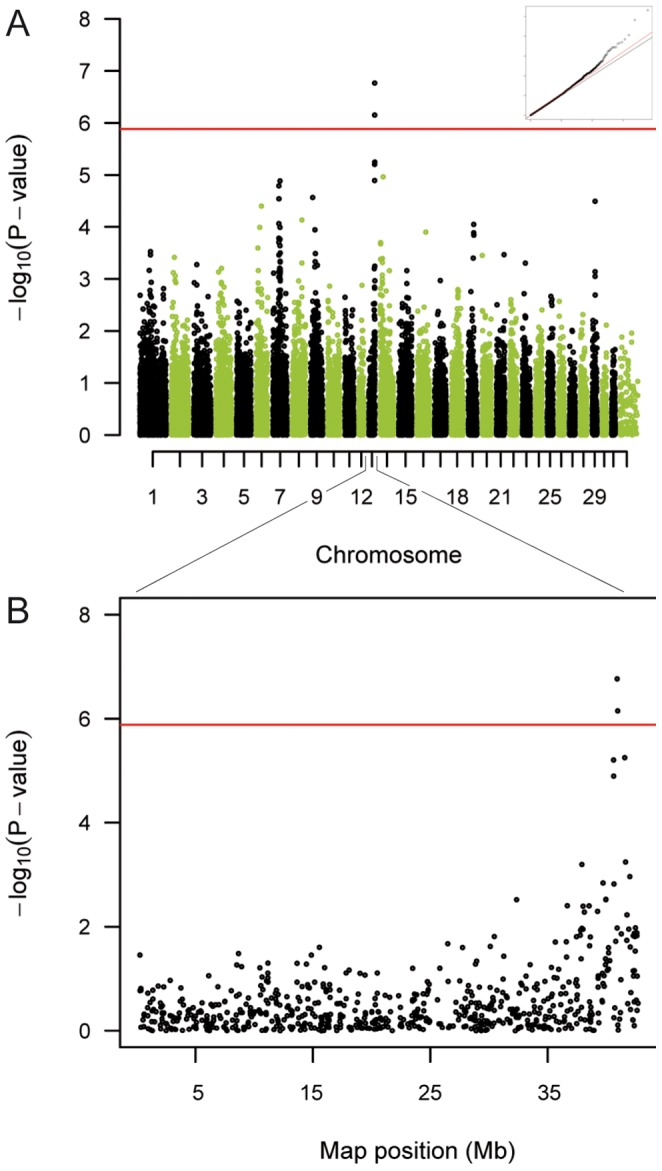
Manhattan plot for maxillary prognathism. (A) A genome-wide case-control study showed a significant association of the phenotype maxillary prognathism on the distal end of chromosome ECA 13. The red line indicates the Bonferroni-corrected significance level (p<1.31×10^−6^). The inset shows a quantile-quantile (qq) plot with the observed plotted against the expected p-values. (B) Two SNPs located towards the distal end of chromosome ECA13 at ∼40.8 Mb were found to be associated with the trait.

**Figure 3:**
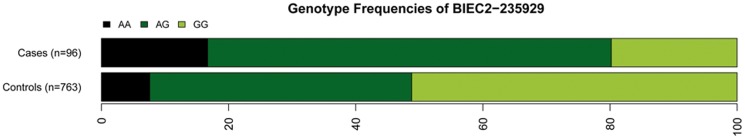
Genotype frequencies of the best associated SNP BIEC2-235929. The genotype distribution indicates a dominant or additive effect of the trait-associated variant.

We initially selected a representative sample set of 1,151 FM horses from the active breeding population and obtained their genotypes at 54,602 SNPs. We excluded 213 horses without information on phenotype and 5 horses with mandibular prognathism. After quality filtering, 859 horses and 38,124 SNPs remained for the final analysis. We performed a case-control allelic association analysis for maxillary prognathism with 96 cases and 763 controls. We analyzed the data using a mixed-model considering the genomic relationships in order to account for population stratification, which resulted in a genomic inflation factor of 1.067 after the correction. Two SNPs within a region on ECA 13 reached the Bonferroni corrected genome-wide significance level - p_BONF_ < 1.31×10^−6^ (Figure 2, Table 1). The two significantly associated SNPs are in complete linkage disequilibrium (r^2^ = 1.00). We also estimated a p-value cut-off for a given false discovery rate (FDR) of 5%. The p_FDR_ was found < 2.03×10^−6^, and thus, only slightly differs from the above mentioned p_BONF_. No additional chromosomal regions reached significance level. The genotype frequencies of the best associated SNP (BIEC2-235929) are illustrated in figure 3 and indicate a dominant or additive effect of the trait-associated variant. This is in agreement with the likely mode of inheritance modeled in PLINK v1.07 [26], where the data fitted best to a dominant model of inheritance. Among the 96 affected maxillary prognathism cases 77 horses (80%) were either homozygous or heterozygous for the risk-allele. However, the other 19 maxillary prognathism cases (20%) were homozygous for the protective allele. Among the 763 FM controls, only 58 horses (8%) carried the risk-allele in homozygous state and 314 controls (41%) were heterozygous for the risk-allele.

**Table 1:** SNPs associated with maxillary prognathism using a mixed-model approach.

SNP name	Equine chromosome	Equine position	Alleles (freq.)^a^		p-value^b^^,^ c
		(EquCab 2)	cases	controls	
BIEC2-235929	13	40,775,412	A (0.48)/G	A (0.28)/G	2.57×10^−7^
BIEC2-235935	13	40,821,697	A (0.47)/G	A (0.28)/G	9.31×10^−7^

a frequency of the minor allele.

b corresponding list of p-values of 1-d.f. (additive or allelic) test for association between SNP and trait; the Bonferroni-corrected threshold for a 5% genome-wide significance level is p_BONF_ = 1.31×10^−6^.

c The two SNPs were in perfect linkage disequilibrium. The small differences in allele frequencies and p-values result from missing genotype calls in a few animals.

### Gene content of the associated region

The associated region is gene-rich. The NCBI annotation of the EquCab 2 assembly currently lists 31 genes and loci in the 500 kb interval from 40.5 Mb to 41 Mb on ECA 13. As the horse genome annotation is still incomplete we inferred the most likely gene content from the human annotation. The NCBI annotation of the corresponding interval in the human genome contains 44 genes and loci (build 37.1, chr16:1,839,753-2,379,239, Table S1). To the best of our knowledge none of these genes and loci has a known role in jaw morphology or bone development. The best associated SNP is located in the *SLC9A3R2* gene that encodes a putative interacting protein of a renal and intestinal Na^+^/H^+^ exchanger (SLC9A3). The second significantly associated SNP is located in the *TBL3* gene encoding transducing (beta)-like 3, a member of the transducing-like (WD40) superfamily. As linkage disequilibrium in the FM horse population extends farther than in humans [1], [2], we consider all 44 genes in the associated 500 kb interval as positional candidates.

## Discussion

We carried out a case-control association analysis for maxillary prognathism with 96 cases and 763 controls using 38,124 SNPs. This analysis led to the identification of a region on ECA13 associated with maxillary prognathism. The identified region is located towards the distal end of chromosome ECA13, with two significantly associated markers at 40,775,412 and 40,821,697 bp. It includes 44 possible positional candidate genes. None of the recently reported candidate genes including *MATN1* are located within this region [24]. Thus, the detected association signal on ECA13 suggests that an additional, currently unknown major gene is involved in the genetics of maxillary prognathism. According to our knowledge, the genetics of prognathism in livestock has not yet been investigated in depth, and only markers from candidate gene studies have been reported so far [24]. To our knowledge this is the first genome-wide association analysis for maxillary prognathism in horses.

Mandibular prognathism has been studied in humans and nine chromosomal regions potentially linked to prognathism have been identified by genome-wide linkage analysis [27]. However, the findings are controversial, suggesting various inheritance patterns for prognathism including autosomal-recessive inheritance, autosomal-dominant inheritance, dominant inheritance with incomplete penetrance or a polygenic model of transmission. The latest results in humans have reported an autosomal-dominant inheritance with incomplete penetrance [27]–[29]. These heterogeneous findings demonstrate that mandibular prognathism follows a complex inheritance pattern, where several loci are involved [23].

Our findings that approximately 80% of horses affected with maxillary prognathism carry the risk-allele on the distal end of chromosome ECA13 indicate that this is a major, but not the only genetic risk factor of prognathism in this breed. The lack of other significant association signals further suggests that maxillary prognathism in FM horses is genetically complex and that other additional genetic risk factors of smaller effect size cannot be excluded. However, the currently available sample size and SNP density limits the detection of additional loci involved in maxillary prognathism. Thus, more samples and/or a higher SNP density will be needed for future studies.

In conclusion, our study identified a novel locus associated with maxillary prognathism in horses. Further research is needed to determine the underlying causal variant(s), which might be used in the future to implement marker-assisted selection and a targeted breeding program against this undesired trait. It will also be of interest to compare our findings with data of other equid and mammalian species, notably cattle and small ruminants, in order to test for potential orthology of the detected region on ECA 13. These findings might ultimately lead to the identification of a previously unknown regulator of jaw morphology.

## Materials and Methods

### Ethic statement

All animal work in this study was conducted in full accordance with the national rules and regulations for animal protection and welfare (paragraph 18 of the Swiss animal protection and welfare legislation). Permission for animal work was given by the Swiss Federal Veterinary Office with a permit to the Swiss National Stud Farm (no. VD 2227.1). The routine collection of blood samples is considered a very low stress in animal research. Thus, apart from the official permission for the animal work performed in this study, no further approval from an ethics committee was needed. Sample collection was performed by state approved veterinarians (see also Signer-Hasler et al. 2012 [10]).

### Animals and phenotypes

The study was conducted with a sample set of 1,077 FM horses. The dataset is described in detail in Signer-Hasler et al. 2012 [10]. We obtained phenotype information of maxillary prognathism during blood sample collection, which was part of a standardized clinical examination of all sampled horses. The clinical examination consisted of a thorough inspection of the horse's integument including the skin, mane, tail and hooves and an examination of the horse's dentition, tendons, joints and musculature. If maxillary prognathism was detected by the examining veterinarian, the horse was classified as affected by the phenotype maxillary prognathism. Visual classification (affected vs. unaffected) was validated in an independent sample of 60 horses by comparing visual classification to measurements in mm derived from incisor imprints in commercially available plastic mold – “modeling clay” (Staedtler® Noris Club® aquasoft Knete Großblock). Since head position can influence the relative position of the upper to the lower jaw, comparison of visual classification to measurements of incisor imprints were performed at three head positions (lowered, neutral and raised). Of the 1,077 horses, 763 were without prognathism, 96 with maxillary prognathism, 5 with mandibular prognathism and 213 without information regarding the prognathism phenotype.

### Genotyping and quality control

We collected EDTA blood samples and isolated genomic DNA from 1,151 horses. The DNA samples were genotyped with the Illumina Equine 50K SNP BeadChip containing 54,602 SNPs. We used the PLINK v1.07 software for pruning of the genotype data set [26]. We removed 48 out of 1,151 genotyped FM horses due to sample duplication. Of the remaining 1,103 FM horses, we removed 10 horses as they had genotype call rates below 90%. Out of the 54,602 markers on the array we removed 12,738 SNPs with minor allele frequencies below 5%, 2,191 SNPs with more than 10% missing genotypes, and 2,730 SNPs strongly deviating from Hardy-Weinberg equilibrium (HWE, p ≤ 0.0001). We calculated the pairwise identity by descent (IBD) from the remaining SNPs and compared them with the corresponding pedigree numerator relationships calculated with CFC [30]. We further excluded 16 animals due to inconsistencies between the marker-based relationship and the pedigree-derived relationship. Thus, the final data set consisted of 1,077 horses (212 males and 865 females) and 38,124 autosomal SNPs.

### Genome-wide association study

We performed an allelic case-control genome-wide association study for maxillary prognathism (96 cases and 763 controls) using a mixed-model approach considering the relatedness of the horses as implemented in the function mmscore in the R package GenABEL [31]. We examined QQ-plots for inflation of small p-values hinting at false positive association signals. After correction for the population stratification the genomic inflation factor was 1.067. We considered genome-wide significance where p-values were below the 5% Bonferroni-corrected threshold for 38,124 independent tests (p_BONF_ < 1.31×10^−6^). The p-value cut-off for a given false discovery rate of 5% (p_FDR_) was determined with the software QVALUE v1.0 [32]. Linkage disequilibrium values between significantly associated SNPs were calculated using PLINK v1.07 [26]. In addition, we performed a case-control study using the software PLINK v1.07 [26] and EMMAX [33]. As the same SNP showed the highest association with maxillary prognathism using PLINK v1.07 and EMMAX, the results of these analyses are not shown.

### Candidate gene analyses

We used the EquCab 2 assembly for the horse genome and build 37.1 for the human genome. We determined horse-human correspondences in the associated interval by using the pre-computed alignments available at the UCSC genome browser. Gene annotations were downloaded from NCBI MapViewer.

## Supporting Information

Figure S1 **Difference in jaw length at the incisor occlusal surface, determined by mold-imprint measurements taken in a lowered head position of horses with (affected) and without (unaffected) visual evidence of maxillary prognathism.**
(DOCX)

Figure S2 **Difference in jaw length at the incisor occlusal surface, determined by mold-imprint measurements taken in a neutral head position of horses with (affected) and without (unaffected) visual evidence of maxillary prognathism.**
(DOCX)

Figure S3 **Difference in jaw length at the incisor occlusal surface, determined by mold-imprint measurements taken in a raised head position of horses with (affected) and without (unaffected) visual evidence of maxillary prognathism.**
(DOCX)

Table S1 **Gene annotation of the human genome in the corresponding segment to the associated horse genome region.**
(XLSX)
