# Supplementary material for: A Chromosomal Region on ECA13 Is Associated with Maxillary Prognathism in Horses
Source: PLoS One. 2014 Jan 21;9(1):e86607. doi: 10.1371/journal.pone.0086607 (PMC3897735; doi:10.1371/journal.pone.0086607)

Measurement in [cm]

affected

unaffected

***Supplementary Figure S2:*** **Difference in jaw length at the incisor occlusal surface, determined by mold-imprint measurements taken in a neutral head position of horses without (unaffected) and with (affected) visual evidence of maxillary prognathism.** Differences in medians between unaffected and affected horses were found significant, with an alpha-level set to ≤0.01. The test statistics were performed using a Mann-Whitney U test and the NCSS software package.

*The median measurement for unaffected horses was 0.0 cm (minimum = -0.1 cm and maximum = 0.4 cm) and for affected horses 0.3 cm (minimum = -0.1 cm and maximum = 0.9 cm), respectively. The corresponding z-value was -4.4.*

Corresponding illustration of head position while making mold-imprint:


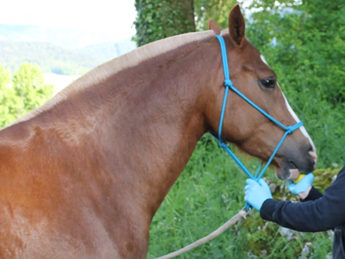

Supplement: Figure S2 — Difference in jaw length at the incisor occlusal surface, determined by mold-imprint measurements taken in a neutral head position of horses with (affected) and without (unaffected) visual evidence of maxillary prognathism. (DOCX) [file pone.0086607.s002.docx]
