# Supplementary material for: A Chromosomal Region on ECA13 Is Associated with Maxillary Prognathism in Horses
Source: PLoS One. 2014 Jan 21;9(1):e86607. doi: 10.1371/journal.pone.0086607 (PMC3897735; doi:10.1371/journal.pone.0086607)

affected

unaffected

Measurement in [cm]

***Supplementary Figure S3:*** **Difference in jaw length at the incisor occlusal surface, determined by mold-imprint measurements taken in a raised head position of horses without (unaffected) and with (affected) visual evidence of maxillary prognathism.** Differences in medians between unaffected and affected horses were found significant, with an alpha-level set to ≤0.01. The test statistics were performed using a Mann-Whitney U test and the NCSS software package.

*The median measurement for unaffected horses was 0.2 cm (minimum = -0.2 cm and maximum = 0.5 cm) and for affected horses 0.4 cm (minimum = 0.1 cm and maximum = 1.2 cm), respectively. The corresponding z-value was -4.4.*

Corresponding illustration of head position while making mold-imprint:


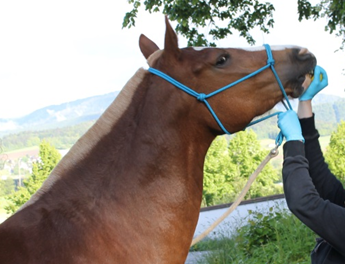

Supplement: Figure S3 — Difference in jaw length at the incisor occlusal surface, determined by mold-imprint measurements taken in a raised head position of horses with (affected) and without (unaffected) visual evidence of maxillary prognathism. (DOCX) [file pone.0086607.s003.docx]
